# Identifying predictive signalling networks for Vedolizumab response in ulcerative colitis

**DOI:** 10.1007/s00384-022-04176-w

**Published:** 2022-05-11

**Authors:** Amrinder Singh, Christopher G. Fenton, Endre Anderssen, Ruth H. Paulssen

**Affiliations:** 1 Clinical Bioinformatics Research Group, Department of Clinical Medicine, Faculty of Health Sciences, UiT- The Arctic University of Norway, Tromsø, Norway; 2 Genomics Support Centre Tromso, UiT- The Arctic University of Norway, Department of Clinical Medicine, Faculty of Health Sciences, UiT- The Arctic University of Norway, Tromso, Norway

**Keywords:** T-cell receptor signalling, Ulcerative colitis, Immune regulation, Network connectivity, Therapy response, Vedolizumab, Infliximab, Signalling pathway, Personalised therapy

## Abstract

**Background:**

In ulcerative colitis (UC), the molecular mechanisms that drive disease development and patient response to therapy are not well understood. A significant proportion of patients with UC fail to respond adequately to biologic therapy. Therefore, there is an unmet need for biomarkers that can predict patients’ responsiveness to the available UC therapies as well as ascertain the most effective individualised therapy. Our study focused on identifying predictive signalling pathways that predict anti-integrin therapy response in patients with UC.

**Methods:**

We retrieved and pre-processed two publicly accessible gene expression datasets (GSE73661 and GSE72819) of UC patients treated with anti-integrin therapies: (1) 12 non-IBD controls and 41 UC patients treated with Vedolizumab therapy, and (2) 70 samples with 58 non-responder and 12 responder UC patient samples treated with Etrolizumab therapy without non-IBD controls. We used a diffusion-based signalling model which is mainly focused on the T-cell receptor signalling network. The diffusion model uses network connectivity between receptors and transcription factors.

**Results:**

The network diffusion scores were able to separate VDZ responder and non-responder patients before treatment better than the original gene expression. On both anti-integrin treatment datasets, the diffusion model demonstrated high predictive performance for discriminating responders from non-responders in comparison with ‘nnet’. We have found 48 receptor-TF pairs identified as the best predictors for VDZ therapy response with AUC ≥ 0.76. Among these receptor-TF predictors pairs, FFAR2-NRF1, FFAR2-RELB, FFAR2-EGR1, and FFAR2-NFKB1 are the top best predictors. For Etrolizumab, we have identified 40 best receptor-TF pairs and CD40-NFKB2 as the best predictor receptor-TF pair (AUC = 0.72). We also identified subnetworks that highlight the network interactions, connecting receptors and transcription factors involved in cytokine and fatty acid signalling. The findings suggest that anti-integrin therapy responses in cytokine and fatty acid signalling can stratify UC patient subgroups.

**Conclusions:**

We identified signalling pathways that may predict the efficacy of anti-integrin therapy in UC patients and personalised therapy alternatives. Our results may lead to the advancement of a promising clinical decision-making tool for the stratification of UC patients.

**Supplementary Information:**

The online version contains supplementary material available at 10.1007/s00384-022-04176-w.

## Background

Ulcerative colitis (UC) is a multifaceted, chronic, immune-mediated inflammatory disorder. UC exhibits inflammation in the mucosa and submucosa, ranging from the rectum, and can spread to proximal segments of the colon [1–4].The patients may undergo periods of remission and relapses [5]. The immunopathogenesis of UC features exaggerated immune response inducing epithelial damage, microbial dysbiosis, abnormal activation of lymphocytes, and infiltration of innate immune cells [6]. The aetiology of UC is multifactorial and potentially caused by genetic, immunological, microbial, and environmental factors [3, 6]. Given the nature of the disease aetiology, there is no single effective therapy for all UC patients. Thus, the use of ineffective UC therapies for moderate-severe cases constitutes a significant burden on the healthcare system [7, 8].

Standard conventional therapies for UC are sulfasalazine, mesalazine (5-ASA), and corticosteroids for the mild-to-moderate disease activity. Some UC patients are unresponsive or intolerant to the standard therapies [4, 9–11] which have prompted the development of new drugs that target tumour necrosis factor (TNF), leukocyte adhesion, JAK-STAT pathway, IL-12 and IL-23, T-helper cell (Th)-1 polarisation, or T-cell activation [12, 13]. UC immunopathogenesis involves the altered immunoregulatory activity by crosstalk between T cell subsets that modulate inflammation [6, 14]. An example of T-cell directed therapy for IBD is a gut-selective anti-α4/β7 integrin heterodimers monoclonal antibody, Vedolizumab (VDZ). Integrin α4/β7 is expressed on immune cells such as T-cell(s). VDZ selectively inhibits the adhesion of integrin α4/β7 to the mucosal vascular address in cell adhesion molecule 1 (MAdCAM-1) which is expressed in the lamina propria [15–18]. Targeting integrin α4β7 prevents the influx of T-cells to the lamina propria, thereby suppressing the gut inflammation [19–21].

VDZ can be used as primary biologic therapy after failure of the standard therapy and also as a secondary therapy for UC patients showing primary non-response, loss of response, or intolerance to anti-TNFα (Infliximab) therapy. It can also be used for the maintenance of clinical remission and is considered a safer yet less efficacious alternative to infliximab [22]. VDZ reduces inflammation in the gut tissue as the gut expresses vascular cell adhesion molecule 1 (MADCAM1) and vascular cell adhesion molecule 1 (VCAM1) molecules [23, 24] In contrast anti-TNFα is associated with systemic immunosuppression [25]. About 30% of UC patients fail to respond to VDZ and suffer tissue damage, and leukocyte-driven inflammatory activity which is associated with TNF-dependent pathways [15]. Other targeted treatment alternatives are Ustekinumab and Tofacitinib. Ustekinumab is a monoclonal antibody biologic targeting both IL-12 and IL-23 to reduce chronic inflammation [26] while Tofacitinib works as an inhibitor which targets the JAK-STAT pathway by inhibiting phosphorylation and activation of JAKs to decrease the inflammatory response [27]. Ultimately, non-responders may require surgical interventions [28]. Therefore, it is important to identify non-responding patients as early as possible during disease development to provide better therapy alternatives.

Our major objective is to seek patient-specific networks that separate VDZ treatment responders and non-responders. In our recent work, we could successfully stratify infliximab responder vs non-responder patients using cytokine signalling network diffusion rates [29]. In this study, we implemented a diffusion model to discriminate UC patients (VDZ responders vs non-responders) to construct patient-specific subnetworks.

## Methods

### Data source

To identify VDZ related studies, a public dataset search on Gene Expression Omnibus (GEO) was performed using the keywords ‘Vedolizumab’ and ‘Ulcerative colitis’. The only hit found was GSE73661 containing Affymetrix GeneChip Human Gene 1.0 ST arrays of UC patients before and after treatment with VDZ or IFX, and non-IBD controls for 178 samples [17]. This dataset contains patients who were recruited from two phase 3 VDZ trials (GEMINI & LTS) (Table 1). Biopsies were taken at week (W) 0 before, and W6, W12, and W52 after VDZ treatment giving a total of 124 colonic mucosal biopsies. The sampling location includes UC left-sided colitis/pancolitis biopsies collected at the edge of ulcers (if present) or at the most inflamed colon segment (absence of ulcers). The colonic biopsies for histological healing assessment were scored using the Geboes index [30, 31]. For endoscopic healing assessment, Mayo subscore was used [32] before and after treatment. VDZ-treated UC patients were classified as responders (n = 14) and non-responders (n = 27) (Table 2) based on the colonic healing sub-score of Geboes index for histological assessment.In this study, patients treated with VDZ all had a previous history of treatment with IFX. We used 112 VDZ-treated patient samples and 12 non-IBD control samples in the analysis. In this dataset, 27 patients did not respond to therapy (non-responder) while 14 had responded (responder) to VDZ therapy (Table 2). We also used a publicly available RNAseq dataset (GSE72819) of another anti-integrin biologic (Etrolizumab) containing UC patients with baseline biopsies (Table 4). This dataset contains a total of 70 samples with 58 non-responder and 12 responder UC patient samples but no non-IBD controls.

**Table 1:**
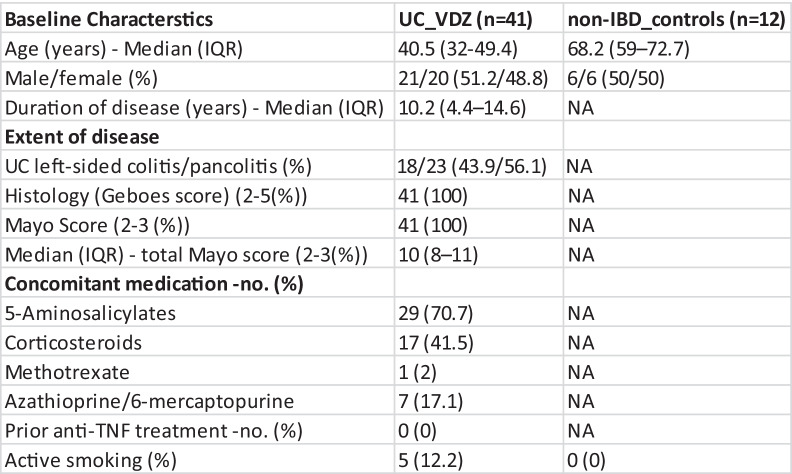
Baseline characteristics of the patient treated with vedolizumab *

*Data adapted from Arijs et al*.* 2018 [17]

### Treatment groups classification

Samples were classified into pre-resistant, post-resistant, pre-responder, and post-responder groups (Table 2). Samples collected at week (W) 0 from patients who did not respond to VDZ at W6, W12, and W52 were labelled as VDZ pre-resistant. Samples collected at W6, W12, and W52 from patients who did not respond to VDZ were labelled as VDZ post-resistant. Samples collected at week 0 from patients who did respond to VDZ at W6, W12, and W52 were labelled as VDZ pre-responders. Samples from patients who did respond to VDZ at W6, W12, and W52 were labelled as VDZ post-responders. Response in this context refers to endoscopic healing at any of the after-treatment time points (W6, W12, and W52) [17].

### Gene expression data pre-processing

Data pre-processing was done using quantile normalisation. We annotated the gene expression matrix with matching gene symbols available in the metadata of the Affymetrix platform (https://doi.org/10.18129/B9.bioc.hgu133plus2.db). Non-coding genes, e.g. microRNAs, pseudogenes, and lncRNAs, were filtered from gene expression data. The final expression matrix contains row values with gene symbols and columns with patients’ sample IDs.

### Transcription factor (TF) identification

We used pandaR [33] to identify important TFs that potentially regulate the gene expression in the UC. The gene IQR (geneIQR > 0.30) was calculated on the gene expression to remove genes with low variance across the samples. To identify the TFs using pandaR, we input protein–protein interaction information using comPPI [34] and regulatory circuits [35] for the regulatory motif binding information in several tissues and cell types. The motif binding set was retrieved from regulatorycircuits.org representing CD4 and CD8 immune cells as relevant T-cell specific immune responses. We selected CD4 and CD8 regulatory circuit because it contains cell types which are surface markers for T-cell. We used the threshold for pandaR result with a score cutoff > 0.01. The result is obtained from pandaR containing TF-gene target edge scores which define confidence for each TF regulating the corresponding target gene. To test which TFs significantly regulate gene expression, a null distribution of TF edge weight was calculated by randomising the TF-gene target 512 times and TFs were selected with an empirical p-value [36] (p < 0.05).

### Generating signalling network

We used a previously constructed diffusion model that estimates network connectivity between receptors and TFs through a signalling network. For creating a signalling network, we used gene expression data, TFs, and receptors. A list of 80 receptors was used which includes IBD GWAS risk genes, cytokines-, chemokines-, pattern recognition-receptors as well as adhesion molecules (Table S1 and Figure S1). To select genes specific to cell surface receptors signalling pathway, we used GO term (GO:0007166) which limits the network to known receptors. Thus, we obtained 24 IBD-relevant cell-adhesion receptors and 37 cytokines receptors with a total of 61 extracellular signalling molecules (Table S1 and Figure S1). By applying significance testing with empirical p-value < 0.05, 34 key TFs were obtained using the sum of their regulatory network edge weights of a total of 643 TFs from pandaR [33]. A complete list of TFs selected for further analysis is provided with their annotations and target genes (Table S2). Genes such as TP53, HSP’s, UBC which have a high number of protein–protein interactions were removed to reduce the complexity of the global network. To further restrict the network, we used GO terms which are associated with cell surface receptor signalling pathway, cytokine-mediated signalling pathway, and integrin α4β7 complex pathway (Table S3). Thus, the final signalling network contains only 309 nodes and 2645 edges for T-cell specific receptors, signal transducer proteins, and TFs.

### Diffusion model

We used our previously published diffusion model [29] which uses gene expression data to generate edge-weighted signalling network graphs for quantifying connectivity from receptors to TFs for each patient. The edge weight is calculated by the product of the gene expression levels of the two genes connected by an edge. The signalling network was used to generate t_50_ variable with 2074 receptor-TF pairs for all samples. t_50_ is defined as the number of time steps needed to reach 50% of the maximum signal received by the TFs over time. The obtained t_50_ data matrix contains scores for each receptors-TF pair per sample representing sample-specific network connectivity. For each gene, a faster t_50_ (low t50 score) means that more signal is being transduced to the connected gene by connected genes.

### Subnetwork identification

The subnetwork is a simplification of the entire global network. The subnetwork is created by shortest paths (using t_50_ values of a gene) between the receptor and the ten top TFs. Note that for each gene, a lower t_50_ indicates a greater accumulation of diffused signal. The top ten receptor-TF pairs (Table S4) all have an AUC > 0.78 (VDZ response vs non-response). The igraph (https://igraph.org/) package was used to plot the subnetworks. For each subnetwork, the sum of the gene t_50_ values for each branch (receptor to TF) for each sample was calculated. The group branch sums were used to compare the groups.

### Statistical analysis

Statistical significance for differentially expressed genes was performed using linear modelling. Multiple testing correction was done with the method of Benjamini and Hochberg [37]. Exploratory data visualisation was done using principal component analysis (PCA). ‘nnet’ a deep learning-based method was used [38] with tenfold cross-validation repeated 20 times using average accuracy to select the final model. Prediction results were evaluated by area under the receiver operating curve (AUC). GO enrichment analysis was performed using the clusterProfiler, Bioconductor package [39]. For comparing the responder and non-responder sample groups, we used Wilcoxon test which is then evaluated using p-value measure.

## Results

### Testing VDZ-specific gene expression

We first assessed four VDZ-specific genes such as MADCAM1, VCAM1, Integrin Subunit Beta 4 (ITGB4), and Integrin Subunit Beta 7 (ITGB7) which are pivotal players in the VDZ drug inhibition of the interaction between α4β7 integrin on T cells with MAdCAM1. To test if these genes can predict VDZ response, we used a linear model on the four integrin-specific genes. Only VCAM1 obtained a significant *p*-value (0.003, AUC = 0.68) and no significance was found in the other three genes (Table 3).

**Table 2:** VDZ response dataset including the number of controls, responder, and non-responder patients. The patient samples were classified into pre-resistant, post-resistant, pre-responder, and post-responder groups at the given time of biopsy of W0 and W (6–52), respectively

**Patient classification**	**Number of patients (n = 41)**
Non-responder patients • Pre-resistant (patient biopsies) at W0 • Post-resistant (patient biopsies) at W (6–52)	**27** 32 49
Responder patients • Pre-response (patient biopsies) at W0 • Post-response (patient biopsies) at W (6–52)	**14** 09 22
Controls	**12**

**Table 3:**
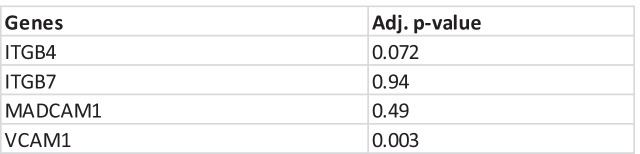
Adjusted p-value of VDZ-specific genes calculated using linear modelling

**Table 4:**
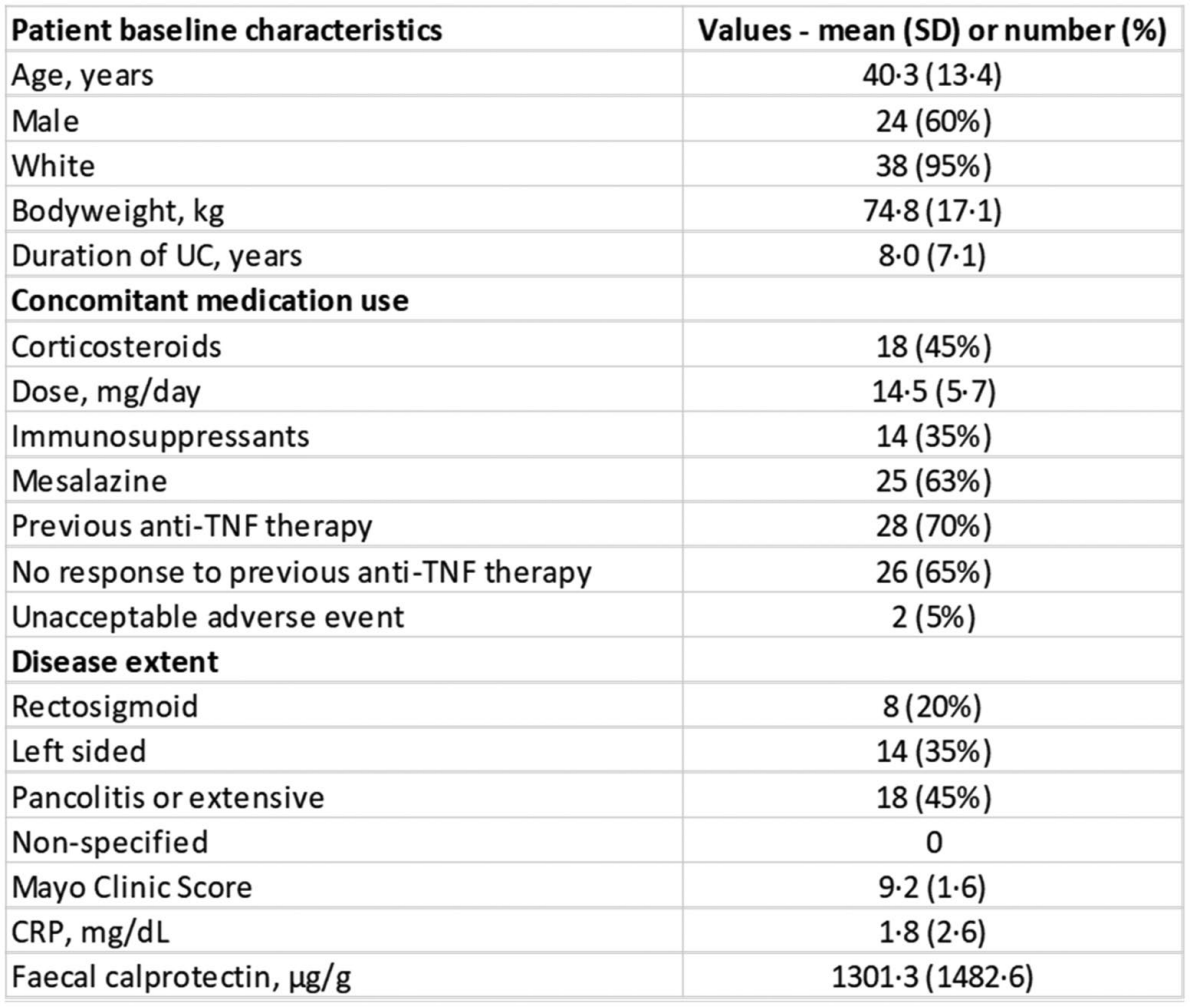
Baseline characteristics of the patient treated with etrolizumab*

^*^ Data adapted from reference Tew et al*.* [66]

### Comparison with a reference method

**Fig. 1:**
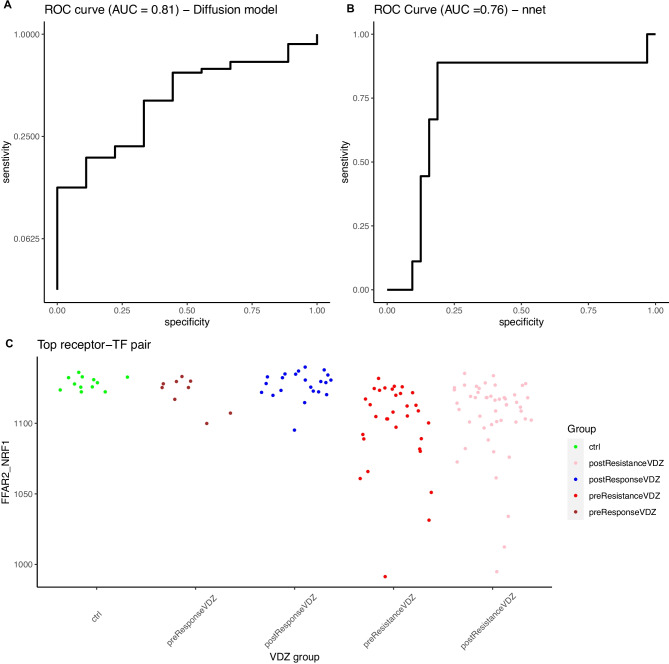
Predictive analysis **(A)** ROC analysis using diffusion model represents AUC = 0.81 for receptor-TF pair (FFAR2-NRF1), which separates treatment responder and non-responder sample groups. **(B)** ROC analysis of ‘nnet’ method represents AUC = 0.76 with a complete gene expression matrix. **(C)** Dot plot represents the differences of t_50_ in the treatment response groups for best discriminant receptor-TF pair FFAR2-NRF1, separating responder pre-and post-treatment, non-IBD controls from the non-responder group. preResponseVDZ, postResponseVDZ represents biopsies obtained at W0 or W (6–52), respectively, from patients that respond to treatment. PreResistantVDZ, postResistantVDZ represents biopsies obtained at W0 and W (6–52), respectively, from patients with non-response

We used a deep learning method, ‘nnet’, a feed-forward neural network algorithm to test the predictive ability to separate treatment responder and non-responder patients. Using ROC (receiver operating characteristic curve), we obtained an AUC of 0.76 for the gene expression (Fig. 1). Similarly, we did a ROC analysis for the diffusion model to compare the predictive ability. We found 39 receptor-TF pairs with AUC > 0.78 (Table S4). The top-scoring discriminators were receptor-TF pairs free fatty acid receptor 2 (FFAR2) nuclear respiratory factor 1 (NRF1), colony-stimulating factor 3 receptor (CSF3R)-RELB, and integrin subunit beta 4 (ITGB4)-ETS proto-oncogene 1, transcription factor (ETS1) with AUC ~ 0.80. These pairs show distinct differences between VDZ responder and VDZ non-responder patients before and after treatment (Fig. 1A and C).

### Comparing gene expression vs. diffusion model

**Fig. 2:**
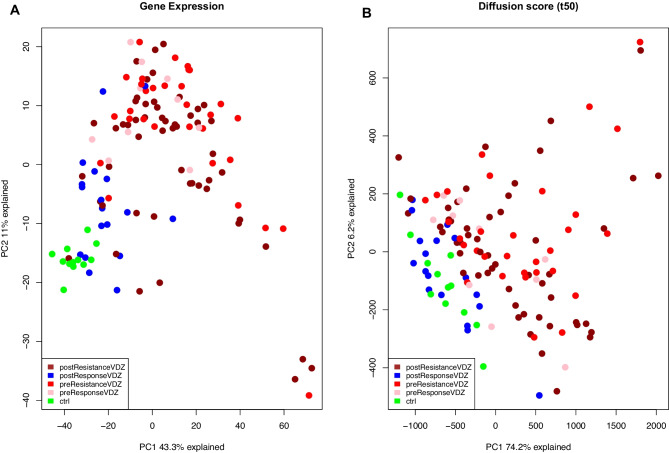
PCA plot showing the difference in UC VDZ treatment response groups. **(A)** Gene expression of non-responder VDZ pre-treatment, Non-responder VDZ post-treatment, responder VDZ pre-treatment, non-responder VDZ post-treatment, and controls. **(B)** Diffusion score (t_50_) of non-responder VDZ pre-treatment, non-responder VDZ post-treatment, responder VDZ pre-treatment, non-responder VDZ post-treatment, and controls

**Fig. 3:**
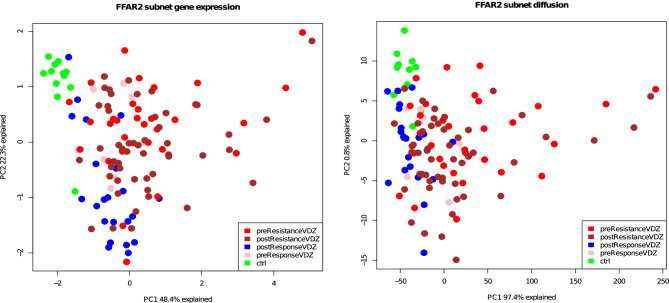
PCA plot on FFAR2 receptor gene on the shortest path between receptor and TFs showing the difference in UC VDZ treatment response groups. **(A)** FFAR2 receptor gene subnet using gene expression of non-responder VDZ pre-treatment, non-responder VDZ post-treatment, responder VDZ pre-treatment, non-responder VDZ post-treatment, and non-IBD controls. **(B)** FFAR2 t_50_ sample branch sums of the shortest paths to top 10 AUC TFs from receptor FFAR2 of non-responder VDZ pre-treatment, non-responder VDZ post-treatment, responder VDZ pre-treatment, non-responder VDZ post-treatment, and non-IBD controls

To compare the predictive ability of the diffusion model against the gene expression data, we used ROC analysis to select the best predictor receptor-TF pairs. Then, we applied PCA on gene expression vs. diffusion model feature score (t_50_) for all VDZ responders, non-responders, and controls samples (Fig. 2A and B). The diffusion model demonstrates an improved predictive power for the separation of patients with VDZ therapy response (see PC1, Fig. 2A) as compared to gene expression. In addition, we applied PCA on receptor FFAR2 which is the best predictor for a subnetwork using the diffusion model result with top receptor-TF AUC score (~ 0.81) and compared it with FFAR2 gene expression (Fig. 3A and B). We found that the PCA of the diffusion model using t_50_ branch sums of the shortest paths could separate VDZ responder and the non-responder group as compared to the gene expression.

### Characterising individualised pathways for treatment response patient groups

**Fig. 4:**
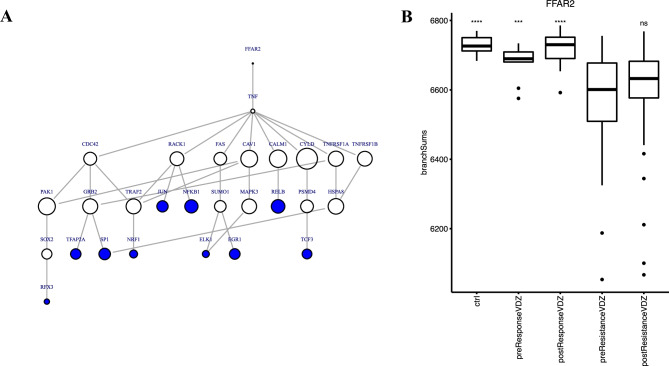
Explanatory analysis **(A)** A simplified subnetwork generated by the shortest paths between FFAR2 to the top 10 AUC TFs. The shortest paths were defined by network diffusion values (t_50_). The size of the node represents the differences between t_50_ between the responder and non-responder groups. A solid black line from FFAR2 to NRF1 represents the shortest path for the top receptor-TF pair FFAR2-NRF1. Red colour indicates receptors, white indicates transducers, and blue colour indicates TFs. **(B)** Box plot shows a separation of treatment response groups using the mean distance of branches in the sub-network. Higher branch length represents slower diffusion of signal in the responder and the non-IBD controls as compared to the non-responder group

Each UC patient exhibits heterogeneity in their network connectivity. Differences in patient-specific networks related to immunological pathways may cause UC pathogenesis. Anti-integrin therapies perturb immunological and inflammatory pathways besides cell trafficking interference [40]. For generating individualised subnetworks, we selected the top 10 receptor-TF pairs (AUC > 0.79), which show the best discriminatory ability (Figure S2). To discriminate VDZ treated responders and non-responders with pre-and post-treatment status, we used t_50_ scores of the diffusion model. We found that diffusion results with the FFAR2 receptor gene to TFs such as NRF1, ETS Like-1 (ELK1), RELB, ETS Like-1 (RFX3) and transcription factor AP-2 alpha (TFAP2A) demonstrate the best discriminatory ability for separating therapy responder and non-responder patients (Fig. 4B and Table S4). To test individual-level differences in the signalling pathway for the patient groups, we generated a simplified version of the overall signalling pathway into the subnetwork describing the diffusion of signal from receptor FFAR2 that passes transducers, to downstream TFs (Fig. 4A). For FFAR2 subnetwork, we selected the top 10 TFs selected with receptor-TF pairs (AUC > 0.77) that separate VDZ responders from non-responder UC patient groups. We found that patient with VDZ non-response exhibits quicker signalling as compared to the patients with VDZ response and controls.

### Identification of patient-specific signalling pathways

**Fig. 5:**
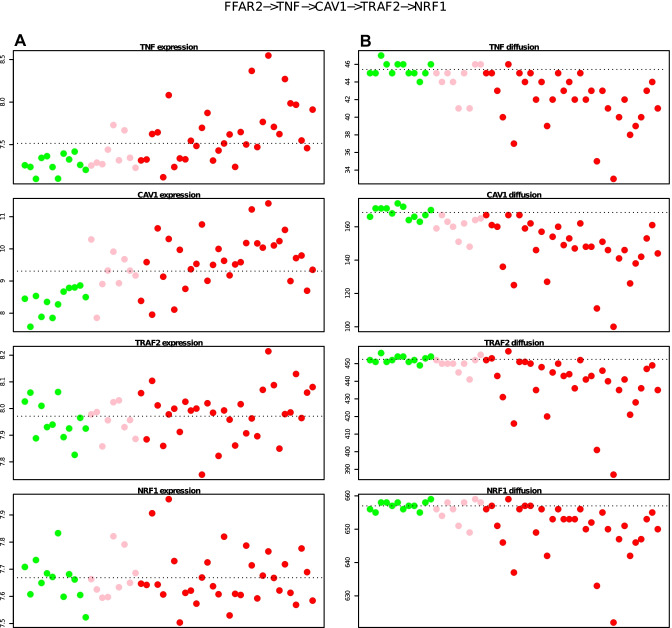
Comparison of gene expression vs. t_50_ diffusion values in path ‘FFAR2-TNF-CAV1-TRAF2-NRF1’ **(A)** Gene expression of shortest path genes. **(B)** t_50_ diffusion values of path genes. The horizontal dotted line represents the mean values in controls. Pre-VDZ responders (pink), pre-VDZ non-responders (red), and controls (green) are indicated

To identify patient-specific signalling pathways, we use the shortest paths in the subnetwork of identified top receptor-TF pairs (Table S4). The top pairs were selected by ROC analysis (Figure S3). Using selected genes in the branch length of the shortest paths, we found the distinct separation of VDZ responders and controls from the non-responders by diffusion model in contrast with gene expression (Fig. 5A and B).

### Testing on the alternate anti-integrin biologic drug (Etrolizumab)

For testing the diffusion model on an alternate anti-integrin biologic, we retrieved the publicly available published RNA seq data (GSE72819) with baseline biopsies from Etrolizumab-treated UC patients (Table 4). This dataset contains 70 samples with 58 non-responder and 12 responder UC patient samples with non-IBD controls. We used the same network generated in VDZ training dataset. We found an acceptable AUC of 0.72 for Etrolizumab dataset (Table S5). Next, we compared the obtained diffusion result with ‘nnet’ prediction, and we found an AUC of 0.69. To check the consistency of the predictive ability of the model on two separate datasets, we applied Pearson’s product-moment correlation test [41] on two sets of AUCs obtained from Vedolizumab and Etrolizumab datasets. We found a significant correlation of 0.68 (95% Confidence interval) with p-value < 0.01.

## Discussion

In this study, we focused on the signalling pathway signatures that stratify VDZ responders from non-responders. UC patients with non-response to VDZ and other biologic therapies have differences in their immune and inflammatory pathways [42]. Using a diffusion model, we generated subnetworks that represent the signalling signatures to discriminate VDZ treatment responders from non-responders. These subnetworks highlight the underlying UC-associated immuno-inflammatory pathways. The diffusion model uses the original gene expression data as well as prior biological knowledge for generating edge-weighted signalling network graphs to delineate the T-cell receptor signalling pathway.

After applying a linear model for gene expression of four pivotal VDZ-specific genes, only VCAM1 was found significantly different between the responder and non-responder patient groups (Table 3). In a previously published study on dextran sodium sulphate (DSS)-induced colitis, Soriano, A. et al. demonstrated the functional role of VCAM1 as a mediator of leukocyte adhesion in colitis and a potent therapeutic effect on immunoneutralisation as compared to MAdCAM-1 and intercellular adhesion molecule 1 (ICAM-1) [43]. Increased expression of VCAM1 in colonic biopsies from patients with IBD is associated with flare-ups leading to disease onset [44]. Gene expression variability is much higher in VCAM1 as compared to MADCAM1. VCAM1 has previously been shown to provide a reliable measure of predicting anti-TNFα therapy response [44].

Exploratory analysis using the diffusion model provides a better stratification of treatment response groups as compared to gene expression alone (Fig. 2). For testing alternate prediction tools for treatment response stratification, we used ‘nnet’ a deep learning algorithm that has recently been utilised for biomarker discovery [38]. We have found that ‘nnet’ and diffusion model features facilitate separation of the VDZ treatment response groups, however, ‘nnet’ fails to outperform the diffusion model (Fig. 1A and B). Here, we can argue that the higher predictive ability of the diffusion model is because of the non-linear transformation of gene expression which is derived from the nature of the signalling network. Another argument could be that the diffusion model enables the inclusion of relevant prior knowledge which explains the underlying biological determinants of VDZ therapeutic response as compared to ‘nnet’ which considers only gene expression.

A previous study has shown that reduction in faecal calprotectin after induction with anti-TNFα treatment, correlates with endoscopic remission. However, the calprotectin level at W0 is a poor predictor of therapy response [45]. Whereas diffusion model could predict the therapy response at W0 using gene expression data, therefore contributing a prognostic value at an early stage of UC.

A recently published study has found distinct signature genes with mucosal gene expression at baseline for VDZ treated UC patients [40]. While comparing these identified genes with the independent VDZ cohort, only about a quarter of the significantly differentially regulated genes were reproducible in the independent cohort. For assessing the model’s reproducibility and generalisability, we used the alternate anti-integrin drug (Etrolizumab) comprising UC patients with treatment responders and non-responder without non-IBD controls. We found 40 best receptor-TF pairs with an AUC > 0.68 with a best receptor-TF pair CD40-NFKB2 (AUC = 0.72) (Table S4). Notably, on both anti-integrin treatment datasets, the diffusion model demonstrated high predictive performance for stratifying responders from non-responders in comparison to ‘nnet’.

Our analysis revealed 48 receptors-TF pairs with AUC > 0.76 that separate the VDZ non-the responders from the responders’ group before and after treatment (Table S4). As expected, the top identified receptor-TF pairs include genes that have a role in regulating intestinal inflammation and involvement in UC pathogenesis. In Fig. 4B, FFAR2 receptor gene to TFs such as NRF1, RELB, early growth response 1 (EGR1), and nuclear factor-kappa B subunit 1 (NFKB1) separates the pre-treatment non-responder vs. responders using branch sum (t_50_) of the shortest path. FFAR2 is a G protein-coupled receptor (GPCR) reported to be a critical precursor of signalling molecules involved in regulating whole-body energy homeostasis, inflammatory and immune responses in the intestine [46, 47]. Non-responder patients have more signals from connected genes that result in quicker signals as compared to the controls and patients in the responder group. The subnetwork obtained using the best receptor-TF pair FFAR2-NRF1 shows the signal from receptor FFAR2 through transducer route 1 (TNF-RACK1-TRAF2) and transducer route 2 (TNF-CAV1-TRAF2) to TF NRF1 (Table S6).TRAF2 is a member of the TNF-receptor-associated factor (TRAF) protein family which directly associates with TNF as a major signal transducer for TNFα-mediated activation of JNK and NFκB [48, 49]. Through NFκB activation, TRAF2 regulates anti-apoptotic signalling by interacting with apoptosis inhibitors [50]. Adjacent to TRAF2 in the signalling network (Fig. 4), RACK1, which is an adaptor molecule that binds to the key signalling molecules involved in the cell migration, integrin adhesion and activity, and T-cell apoptosis [51–54]. A previous study demonstrates the role of RACK1 as a negative regulator of NF-κB signalling, NF-κB-mediated cytokine induction and inflammatory reactions [55]. Next, CAV1 is a gene involved in diverse signalling pathways and plays an essential role in cell proliferation, apoptosis, lipid migration, and exhibits a protective role in intestinal inflammation for IBD [56–58]. We hypothesise that TNF, RACK1, CAV1, and TRAF2 are part of the protein complex in which TNF and TRAF2 are connected to the TNFRSF1A. This might modulate downstream signalling to transcription factor NRF1 (Fig. 4) which is involved in maintaining organ integrity by regulating cytoprotective defences through cellular redox homeostasis [59, 60] by preventing cells against proteasome inhibition through regulation of proteasome gene expression. With a higher accumulation of proteasome inhibitors, NRF1 loses its potency to initiate transcription [61]. Some studies showed a reduction of intestinal inflammation by targeting immunoproteasome that attenuates proinflammatory signalling in DSS-induced colitis study on mice and IBD patients [62–64]. Targeting NRF1-mediated endoplasmic reticulum-associated degradation (ERAD) pathway could increase the therapeutic efficacy of proteasome inhibitor drugs for providing readily actionable targets [65].

## Conclusions

We used a network-based diffusion model to highlight genes and their interactions in signalling pathways which may be predictive in response to the anti-integrin treatment. In our case, the diffusion model outperformed a deep learning method (nnet) and can give comparable prognostic ability at initial diagnosis to longer-term monitoring of calprotectin. The obtained subnetworks feature genes involved in cytokine and fatty acid signalling. The results suggest that anti-integrin drug responses in cytokine and fatty acid signalling pathways can discriminate UC patient populations. As the availability of high throughput RNA sequencing in the clinic increases, these findings may offer useful insights into the development of clinical decision-making to aid in selecting UC treatment strategies.

## Supplementary Information

Below is the link to the electronic supplementary material.

Supplementary file1 (DOCX 16 KB)

Supplementary file2 (XLSX 14 KB)

Supplementary file3 (XLSX 1107 KB)

Supplementary file4 (XLSX 13 KB)

Supplementary file5 (XLSX 18 KB)

Supplementary file6 (XLSX 25 KB)

Supplementary file7 (XLSX 14 KB)

## Data Availability

Public datasets used for analysis in this study are available in the Gene Expression Omnibus (GSE73661, GSE72819) repository.

## Abbreviations

UC: Ulcerative colitis
IBD: Inflammatory bowel disease
TNF: Tumour necrosis factor
IFX: Infliximab
VDZ: Vedozilumab
ETR: Etrolizumab
5-ASA: 5-Aminosalicylic acid
GEO: Gene Expression Omnibus
IQR: Interquartile range
ROC: Receiver operating characteristic
AUC: Area under the ROC curve
GO: Gene ontology
TF: Transcription factor
MAdCAM-1: Mucosal vascular address in cell adhesion molecule 1
VCAM-1: Vascular cell adhesion molecule 1
ICAM-1: Intercellular adhesion molecule 1
ITGB4: Integrin subunit beta 4
ITGB7: Integrin subunit beta 7
lncRNAs: Long non-coding ribonucleic acid
PPI: Protein–protein interaction
FFAR2: Free fatty acid receptor 2
DSS: Dextran sodium sulphate
