# Supplementary material for: Identifying predictive signalling networks for Vedolizumab response in ulcerative colitis
Source: Int J Colorectal Dis. 2022 May 11;37(6):1321–33. doi: 10.1007/s00384-022-04176-w (PMC9167201; doi:10.1007/s00384-022-04176-w)
Supplement: Supplementary file 1 — Supplementary file1 (DOCX 16 KB) [file 384_2022_4176_MOESM1_ESM.docx]

## Supplementary Figures:

**Figure S1:** Outline representing literature curated cytokines, receptors, identified TFs, and constructed signalling network with nodes and edges.

**Figure S2:** PCA plot on 10 top-receptors genes showing the difference in UC VDZ treatment response groups. **(A)** Receptor gene subnet using gene expression of non-responder VDZ pre-treatment, non-responder VDZ post-treatment, responder VDZ pre-treatment, non-responder VDZ post-treatment, and non-IBD controls. **(B)** receptor gene t50 branch sums of shortest paths to top 10 AUC TFs from receptor of non-responder VDZ pre-treatment, non-responder VDZ post-treatment, responder VDZ pre-treatment, non-responder VDZ post-treatment, and non-IBD controls.

**Figure S3:** Comparison of gene expression vs. t50 diffusion values in ten pathways of top ten rec-TF pairs identified using AUC score **(A)** Gene expression of path genes. (**B)** t50 diffusion values of path genes. The horizontal dotted line represents the mean values in controls. Pink represents pre-VDZ responder. Red represents pre-VDZ non-responder, Green represents controls.

Supplementary Table:

**Table S1:** List of literature curated extracellular signals (cytokines, receptors, pandaR identified Key TFs.

**Table S2:** Annotation of Transcription Factors (TFs) obtained in UC containing TF name, TF score, empirical P-value, and TF associated target genes respectively.

**Table S3:** List of GO terms and their molecular functions used for generating signalling network.

**Table S4:** List of top receptor-TF pairs for VDZ dataset with their AUC score (0.76-0.81).

**Table S5:** List of top receptor-TF pairs for ETR dataset with their AUC score (0.68-0.72).

**Table S6:** Table summarizing the evidence of protein-protein interactions for the genes present in the signalling pathway. This includes source protein, target protein, publication (PMID), Interaction type, source databases, Interaction detection methods.
